# Deconstruction and Realization of Sports Entity Simulation Based on Fish Swarm Algorithm

**DOI:** 10.1155/2022/4109170

**Published:** 2022-07-22

**Authors:** Kai Wu

**Affiliations:** School of Public Teaching and Practice, Wuhan Technical College of Communications, Wuhan 430065, Hubei, China

## Abstract

Due to the limitation of sports movement, the current simulation technology of sports entities is prone to deficiencies in capturing dynamic motion figures and is prone to lack of accuracy. It is also affected by external noise and brightness. To solve these problems, this paper proposes a sports entity simulation based on the fish swarm algorithm and compares the figure effectiveness, figure segmentation, core point, and noise reduction effect of the two in the shooting figure. Through the comparison, it is found that the figure is more appropriate to the real moving figure, the motion capture is more accurate, and the number of core points is related to the accuracy of motion capture. The more core points, the more accurate the motion capture, and the noise reduction effect is also increased by 20.3%, which reduces the impact of brightness on the motion simulation. The difference in the effect of the traditional simulation technology (particle swarm algorithm) and the entity simulation based on the fish swarm algorithm was also compared. The combination with the artificial fish swarm algorithm is to simulate the moving entity and learn from some reference data. By comparing the data between the two after the experiment, it is concluded that the fish swarm algorithm is more effective in the simulation of sports entities.

## 1. Introduction

The physical simulation of sports is recorded by sensors, and then the movement information in the space is transmitted to the processing center of the computer and processed by computer to get the basic three-dimensional human body model. On this basis, the human body frame is drawn to realize the physical simulation of sports. At present, the main technologies used to capture sports movements include optical capture, mechanical capture, and electroacoustic capture. Mechanical and electroacoustic capture methods are limited by sports and have a relatively broad acquisition range. However, the accuracy of motion capture is not enough. The advantage of the optical capture method is that it will not be blurred, but the number of captured points is not enough to meet the requirements of moving entities, and the collection location is affected by the brightness, and the equipment itself is also easily affected. It is easy to cause the external light data to interfere with the motion data, resulting in confusion in the analysis of the equipment during operation, and it is more complicated to deal with. Although electroacoustic capture has low operating costs, the capture accuracy is not enough. In this regard, motion capture technology is not satisfactory. In this paper, the simulation calculation of moving entities based on fish swarm algorithm is introduced.

In this paper, based on the fish swarm algorithm, the sports motion detection system is compared. By simulating the irregular images under human body dynamics, through the corresponding tools, under the condition that the background is not set, the influencing factors existing in sports are removed, background, shadows, brightness, noise, and so forth, and the main moving points of the human body under sports are extracted and simulated, which is of great significance to the improvement of simulation technology.

In this paper, a physical simulation of sports based on fish swarm algorithm is proposed. By comparing the figure effectiveness, figure segmentation, core point, and noise reduction effect of the two in the shooting figure, it is found that the figure is more appropriate to the real moving figure, the motion capture is more accurate, and the noise reduction effect is also increased by 20.3%. By comparing the data between the two after the experiment, it is concluded that the fish swarm algorithm is more effective in the simulation of sports entities.

## 2. Related Work

With the gradual acceleration of urbanization, people's quality of life has gradually improved. Every household has begun to prepare a car as a means of transportation, and the problem of urban traffic has gradually become more and more serious, and it is easy to cause congestion. The urban green wave traffic control system is one of the effective means to solve the problem of urban traffic congestion and reduce vehicle delays. In response to the problem of green waves, Ma C investigated the causes of congestion in the city through the fish swarm algorithm and found that the irregular movement of the crowd, the irregular parking of vehicles, and people's traffic awareness are the main reasons for the impact [1]. The progress of industrialization has brought about changes in weather, increasingly serious smog pollution, and a significant decline in air quality. Zhu X proposed a haze prediction method based on multifractal dimension (MFD) and coevolutionary discrete artificial fish swarm algorithm (CDAFSA). Using CDAFSA, MFD, and extreme learning machine (ELM), the algorithm population is initialized through good point set theory; the swarming, chasing, and foraging behaviors are improved by introducing the swimming speed of artificial fish; the algorithm is discretized; and competition and cooperative operation are introduced. Second, the haze dataset is reduced by CDAFSA and MFD; finally, a haze prediction model is established by using ELM. The experimental results show that the proposed prediction method is superior to the traditional method and has high stability and credibility [2]. The accurate prediction of wind power in wind farms is of great significance to the economic operation of wind farms and the safe operation of power grids. The randomness and volatility of wind in wind farms make wind power forecasting more difficult. Based on the characteristics of wind in wind farms, combined with relevant meteorological data such as wind speed, temperature, and humidity in wind farms, Zhai built the ultimate learning machine model (ELM) for wind energy forecasting and established an artificial fish swarm algorithm (AFSA) for rapid optimization. The constructed model is trained and predicted by the actual meteorological data and wind data of the wind farm. The results show that this method has higher predictive ability than the wind power prediction model of particle swarm [3]. The fish swarm algorithm is not only used in green waves, air quality surveys, and wind farms but also gradually used in sports.

Simulation technology, which is essential to virtual reality, has been extensively applied in the study of physical education. This ground-breaking programme has had a profound effect on conventional teaching methods and ways of thinking, and it has created enormous opportunities for the advancement of physical education. Yili examined the use of simulation technology and stimulus-response theory in the field of physical education with a focus on the drawbacks of the conventional physical education teaching method. This served as the foundation for the invention of the teaching method for physical education. The findings demonstrate that auxiliary teaching can enhance students' theoretical and practical understanding of physical education, enhance their academic performance and learning enthusiasm, and serve as a useful benchmark for physical education teaching innovation [4]. The active safety capabilities of cars have evolved into a crucial criterion for assessing their quality as the automotive industry continues to grow. The antilock braking system is an important tool to increase the active safety of the vehicle and prevent the wheels from locking. It does this by adjusting the torque to ensure that the vehicle slips at the ideal rate. It can still turn even when braking hard, cutting down on braking distance and time to lessen tyre wear. Song conducted research on an antilock braking system based on simulation technology on the basis of examining the pertinent principles. A simulation model for the antilock braking system in cars has been developed. It simulates the simulation model. According to the findings, antilock braking systems on vehicles perform better than conventional braking systems at maintaining slip rate, braking distance, and braking time [5]. During the wiring process of complex mechatronic products, the spatial pose and shape of the flexible wiring harness are prone to change. As a result, the sampling information of the wiring harness is inaccurate during the wiring process, which seriously affects the wiring efficiency. Wang proposed a physical property modeling and deformation simulation method of flexible cable harness based on the exact Cosserat model. The physical property model of the wiring harness based on the exact Cosserat model was established, and then the dynamic balance formula of the flexible wiring harness was constructed [6]. Simulation technology is an emerging technology in the field of system modeling and simulation research in recent years, and it has always been the focus of research. According to the simulation technology requirements of battlefield command decision-making, equipment task planning, and equipment maintenance support, the outstanding problems of existing simulation technology are summarized. That is, the domain model is stable and has no self-evolution ability. Ge put forward the concept of equipment parallel simulation technology and pointed out its characteristics. It systematically introduces its theoretical origin and research status of related technologies and discusses the main modeling technologies such as online perception of equipment degradation status and establishment of equipment degradation [7]. Simulation technology has been widely used now and is a milestone technology for the country to enter into rapid development.

## 3. Simulation System Description and Key

### 3.1. Simulation System

**Figure 1:**
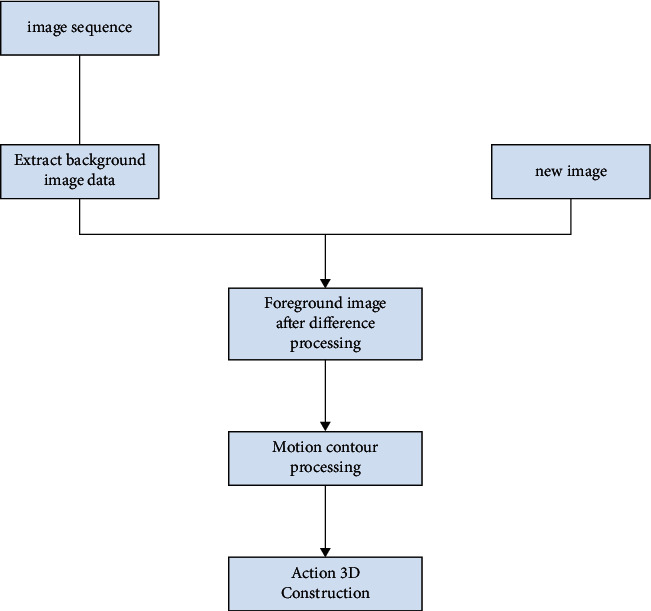
Flowchart of the simulation system.

The simulation system must go through a standard motion figure processing stage before capturing, simulate the dynamic figure data in the computer body, and maintain the background's original state. Additionally, noise interference may get in the way of capturing. The image cannot be captured directly as the background because the brightness in the capture space is also subject to slight variations, which will influence the capture [8]. The flow figure of system processing is shown in Figure 1.

Because the light intensity needs to be processed, it is necessary to extract new data from the background image and replace the previous background image. The replacement condition requires that the previous background image be relatively stable, and there is not much numerical deviation. When replacing, it cannot be said to be a complete replacement but only covers the previous information with part of the information and uses the difference algorithm between the sports image and the replaced background image to obtain the required background image. Through color processing, the image color is converted to the original gray. Then, the image data is differentiated through the threshold algorithm, and differential processing can identify differences in the background and quickly fill in the blanks. The differentiated data is used to complete the empty space in the replacement in a dynamic mathematical way, and a relatively complete three-dimensional map of sports points is obtained. 3D map is the basis of human motion contour and the most basic step of simulation technology. By extracting the edge points of the three-dimensional image [9], it will not be completely closed under normal conditions. By connecting the edge points, the key points of human body movement can be obtained, and the direction or position of other movements can be obtained instantly. The motion data outline will also coincide with the connected line segments. Through the obtained key points, the outline of human motion can be realized by drawing and 3D construction through simulation technology.

### 3.2. Influence Points of the Realization System

#### 3.2.1. Background Extraction

There are many algorithms for background extraction. For example, there are interframe difference method, background difference method, ViBe algorithm, ViBe + algorithm, and so on. In this paper, the traditional fish swarm algorithm and the improved artificial fish swarm algorithm are used. Before extracting the background, the captured image points need to be sorted in a certain order. It is concluded that the sorted image is different from the previous background image and becomes cluttered. In ascending order from small to large, the pixel of the first image is the minimum value in the image. Similarly, the pixel value of the last image is the maximum value. Assuming that all image pixels are *L*, the noise interference is placed next to the background image, and the pixels arranged in the middle are used as the background, which can also prevent the pixels from being too large or small, resulting in the confusion of the entire image. The value of *L* in the system is selected as 9, and then the fifth figure is the background image. For the value of *L*, its size also has certain requirements. If it is too large, it will easily lead to too much calculation and affect the result. If it is too small, it cannot handle noise. Background extraction is the first shot of motion capture, but it cannot be captured as soon as it is turned on. Because of direct capture, the figures returned by the system are inconsistent, or there is a delay, or there are other cases where the pixels are not clear. This needs to be prepared in advance before capturing. When the figure reaches 25 frames, it will gradually stabilize. When the system is running, the previous frames can basically be abandoned directly, and the figures are the main ones, which are stabilized images. The frame rate is also a point to consider, because VC++ is used in this article as an auxiliary tool, the camera is the main sensing instrument, and the control instrument is a computer. Because, during image processing [10, 11], for image capture, the computer processing time is assumed to be *t*, if the *T* value is very large, the processing time of each photo is too long, which is not considered in this article, and does not meet the actual operation purpose. If the value of *T* is small, assuming that it is 0.01 s, it means that the computer needs to process hundreds of figures per second. But, for sports, the magnitude of the change is only a few microseconds, and the images in the vicinity of a few microseconds are similar, and there is not much difference. The general camera frame rate is 20–120 Hz. If the value of *t* is smaller, more images will be processed, and the situation of half-image will easily appear when the computer processes it. Get either the image of the upper half frame or the image of the lower half frame, or even there may be no figure. In order to ensure validity and accuracy, the processing frequency of the computer must be less than the capture frequency of the camera.

#### 3.2.2. Human Motion Detection

There are three methods currently used for detection, the difference detection method [12], the background difference method, and the optical flow method [13]. The difference method is used in this paper. For the difference method, the background image and the previous frame image are determined through the calculation of the difference image, but it cannot be directly used in the background image because the image has color difference [14]. There are many influencing factors in the image, the color difference of the image, the scene of brightness, the number of recorded frames, and so forth, which cannot be directly used as the data information of the movement. The pixels are mainly formed in three ways, and the relationship between them is

(1) $$\begin{array}{c} \text{Gray}=0.3R+0.6G+0.1B. \end{array}$$

In the above formula, *R*, *G*, and *B* are the main paths of pixels. The color of the background is converted into the original gray through color, and the image data is differentiated through the threshold algorithm. Finally, the irrelevant points in the image are eliminated by the erosion algorithm. After adjusting and shrinking the image, the adjusted image is calculated, connected, and filled to supplement the empty points [15].

### 3.3. Motion Profile and Straight Line Fitting

First, the data points of the obtained foreground image are connected by line segments. Because the obtained image is a binary image, the edge points need to be eliminated by tools. Connecting the points whose edges conform to the outline, the pixels slowly form a spatial figure, and these points are saved in ascending order to remove the useless line segments that are spent separately. Finally, the multisegmented lines are formed into the core, and the core is found to realize the fitting. The obtained image data is arranged and combined in space through pixels to form a mathematical problem, which is also conducive to finding the position of pixels. Drawing through spatial coordinate points, in order from small to large, the order of connections is very important for image formation. If the order of connections is wrong, it may not be possible to form the outline of the human body, and you need to start from the first step. Because the contour line segments of the image are not necessarily all closed, there will be some redundant points to form connecting line segments. These useless line segments need to be removed, which can prevent subsequent processing [16]. The traditional simulation technology to realize the influence points of the system is not perfect, and the artificial fish swarm algorithm is introduced to improve the imperfect points.

### 3.4. Artificial Fish Swarm Algorithm

**Figure 2:**
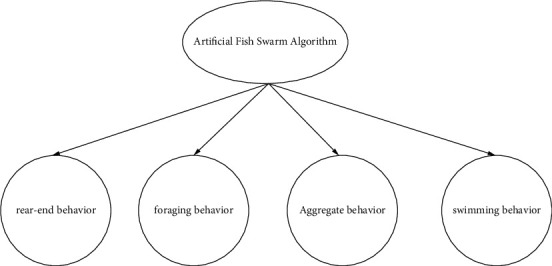
Fish presence status.

Chinese researchers proposed an intelligent calculation technique called the artificial fish swarm algorithm. The algorithm uses the various locations of the artificial fish as potential answers and the amount of food the fish can detect as the importance of the response. By simulating fish swimming, foraging, gathering, and tail-chasing, the best solution can be found.  Figure 2 [17] depicts the various states that fish schools could be in. In this study, cloud theory is applied, cloud learning and cloud variation factors are incorporated into a single artificial fish, and a cloud-based artificial fish swarm algorithm is proposed to address potential issues with artificial fish calculation [18].

In traditional fish swarm algorithm, assuming that the number of fish schools is *S*, the number of experiments is *C*, the fish school detection distance is *D*, the step size is *L*, *θ* is the proportion of fish in the artificial pond, and the interval between each fish is *X*, the food visibility of the artificial fish pond is

(2) $$\begin{array}{c} Q=f\left(x\right). \end{array}$$

In the above formula, *Q* is the value of the correlation function. For the four behaviors of the fish school, the expressions of tail-chasing, foraging, gathering, and swimming are as follows.

As regards rear chasing behavior, when the status of artificial fish breeding is *W*_*i*_, find the most suitable *W*_*j*_ within the detection distance [19], and the proportion of fish in the artificial pool is less than *θ*, and move *W*_*i*_ to *W*_*j*_ by one step. According to the following formula, if not executed, foraging behavior will be performed.

(3) $$\begin{array}{c} W_{i}=W_{i}+L\times \text{rand}\left(\right)\times \frac{W_{j}-W_{i}}{\left\|W_{j}-W_{i}\right\|}. \end{array}$$

Considering foraging behavior, when the state of artificial breeding is *W*_*i*_, the most suitable new state is *W*_*j*_ within the detection distance. If suitable *W*_*j*_ is found, the artificial fish will move to *W*_*j*_; if no suitable *W*_*j*_ is found, then *W*_*i*_ will continue to find the most suitable *W*_*j*_ and move towards *W*_*j*_; after *C* times, if no suitable *W*_*j*_ is found, the following formula is performed:

(4) $$\begin{array}{c} W_{j}=W_{x}+\left(2\text{rand}\left(\right)-1\right)\times D,\\ W_{x}=W_{x}\times \left(1+\left(2\text{rand}\left(\right)-1\right)\right)\times L\text{)}. \end{array}$$

For aggregation behavior, when the status of artificial fish breeding is *W*_*i*_, find the most suitable *W*_*c*_ within the detection distance, and the proportion of fish in the artificial pool is less than *θ*, move *W*_*i*_ to *W*_*c*_ by one step, and gradually form aggregation. If aggregation is not achieved, foraging behavior will be performed [20].

(5) $$\begin{array}{c} W_{i}=W_{i}+L\times \text{rand}\left(\right)\times \frac{W_{c}-W_{i}}{\left\|W_{c}-W_{i}\right\|}. \end{array}$$

For swimming behavior, when the state of artificial breeding fish is *W*_*i*_, and it swims freely within the detection distance, this behavior can be regarded as foraging behavior or free swimming behavior.

From the four modes of fish swarms, the tail-chasing behavior indicates that there is an intersection of answers in the process of finding a solution, the foraging behavior is on the way to find the optimal solution, and the aggregation behavior is that the answer to the optimal solution is very close, and swimming behavior indicates no solution. It can be understood that the traditional artificial fish swarm algorithm strengthens the aggregation of fish swarms but reduces the role of individual fish in the swarm. The free swimming of the fish will lead to a larger range of results in the subsequent algorithm, which in turn affects the foraging behavior of the fish [21]. When seeking the optimal solution, it enters a local circle, which is not perfect enough. Therefore, in this paper, the fish swarm algorithm of cloud theory is combined, and the two are modeled, which is also called Zhengtaiyun. Introduce cloud learning factors and cloud variation factors to improve algorithm learning ability in self-learning algorithms. The improved algorithm will be obtained, by comparing the behavior of the fish before with the individual fish, which can greatly improve the degree of learning identification, reduce the free swimming behavior of fish, enhance the aggregation of the optimal solution, and make it possible to search for the optimal solution more quickly [22]. The density here is the function corresponding to the best solution in the largest fish group. When finding the smallest solution, it can be obtained by the inverse of the maximum value. Assuming that the current position of the artificial fish is (*x*_*i*_, *y*_*i*_) and the density value is (*F*_*x*_, *F*_*y*_), there can be

(6) $$\begin{array}{c} R_{x}=\frac{F_{x}}{F_{x}+F_{y}}\times x_{i}+\frac{F_{x}}{F_{x}+F_{y}}\times y_{i},\\ R_{n}=\frac{D}{L_{1}},\\ H_{e}=\frac{R_{n}}{L_{2}}. \end{array}$$

The cloud variation factor is the transformation of a single fish individual into an individual under the new model under the condition of Zhengtai cloud. Let the current position of the artificial fish be *x*_*i*_ and the density of a single artificial fish be *F*_*x*_, which can be expressed as

(7) $$\begin{array}{c} R_{x}=x_{i},\\ R_{n}=\frac{D}{L_{3}},\\ H_{e}=\frac{R_{n}}{L_{4}}. \end{array}$$

The random distribution certainty of random fish swarms can be achieved through Zhengtaiyun, which is denoted by *G* in the text. This is a vague concept that has a certain relationship with probability. In the improved artificial fish swarm algorithm, the distance of fish with high density is small, so as to obtain a suitable specified value, the range of *G* needs to be dynamically processed by the objective function, and there are two processing methods [23].

Randomness X-condition cloud generator method is expressed as follows:

(8) $$\begin{array}{c} R_{x}=F_{\max}:R_{n}=\frac{F_{\max}-F_{\min}}{L_{5}}:H_{e}=\frac{R_{n}}{L_{6}},\\ R_{n}^{,}=\text{randn}\left(R_{n},H_{e}\right):G=e^{\left(F^{,}-R_{x}\right)/-2R_{n}^{,}\left(F^{,}-R_{x}\right)/R_{n}^{,}}. \end{array}$$

In the X-condition cloud generator, three special values are assigned, which are *R*_*x*_, *R*_*n*_, and *H*_*e*_, and the influence value drop(*x*_0_, *G*_*i*_) is formed.

Deterministic linear function method is expressed as follows:

(9) $$\begin{array}{c} G=G_{\text{max}}-\frac{F_{\text{max}}-F^{,}}{F_{\text{max}}+F_{\text{min}}}\left(G_{\text{max}}-G_{\text{min}}\right). \end{array}$$

In the above formula, *F*_max_ is the maximum fish school fitness, *F*_min_ is the minimum fish school fitness value, *G*_max_ is the maximum fish school density due to external influence, and *G*_min_ is the minimum fish school density. In general, *G*_max_ is within (0.93–1), *G*_max_ is selected as 0.96, and *G*_min_ is selected as 0.19, which is also a conventional value. It can be seen from the above that the fitness value of the individual fish is inversely proportional to the search range. The larger the value, the smaller the range. When the fitness is the largest, *G* = 1. After the processing operation is carried out, it is brought into (7), and the result is unfavorable for population diversity. When *G* is 0.95, according to *R*_*n*_, the value of *L*_5_ is less than 4, which is 3.7. In the normal range, the result of L6 is 5–15, and the value of L6 is 10. Although *R*_*n*_ and *H*_*e*_ play a key role in the model, when multiple calculations are performed, replacement iterations will be performed to replace the previous data, so this has little effect on the values in the following text.

### 3.5. Segmentation of Sports Images Based on Fish Swarm Algorithm

The pixels of the image are arranged in order from low to high, and the image formed by the pixels needs to be segmented to obtain the core points. Then separate the multiple images of the movement, and segment them from the background image. When the color difference between the background image and the required image is obvious, when the computer processes as high values on both sides, this is the best separation period. If the computer processing is not at the highest level, no image segmentation is required. In this paper, threshold image segmentation is used. In this paper, threshold image segmentation is used. Through the thresholds *U* and *U* ∈ (0，*L* − 1), the figure can be divided into two parts, and the background and positioning are *B* and *W*, respectively. Assuming that *P* is the probability of the original gray in Figure 2, *P*_*t*_=∑_*i*=0_^*t*^*p*_*i*_, and then the entropy function is

(10) $$\begin{array}{c} H\left(t\right)=H_{B}+H_{W},\\ H_{B}=-\sum_{i=0}^{t}\frac{p_{t}}{P_{t}}\ln\frac{p_{t}}{P_{t}},\\ H_{W}=-\sum_{i=t+1}^{L-1}\frac{p_{t}}{P_{t}}\ln\frac{p_{t}}{1-P_{t}}. \end{array}$$

When *H*(*t*) is the maximum value, *t*^*∗*^ is the optimal solution. When there are *K* thresholds in the thresholded image, which is *t*^*K*^, the entropy function can be calculated to obtain the maximum value; then there are

(11) $$\begin{array}{c} H\left(t_{1},t_{2},\ldots t_{k}\right)=-\sum_{i=0}^{t_{1}}\frac{p_{i}}{P_{t_{0}}}\text{ ln}\frac{p_{i}}{P_{t_{0}}}-\sum_{i=t_{1}+1}^{t_{2}}\frac{p_{i}}{P_{t_{1}}}\text{ ln}\frac{p_{i}}{P_{t_{1}}}-\cdots \sum_{i=t_{k}+1}^{L-1}\frac{p_{i}}{P_{t_{k}}}\text{ ln}\frac{p_{i}}{P_{t_{k}}}. \end{array}$$

The same as above, when *H*(*t*_1_, *t*_2_,…*t*_*k*_) is the largest, *t*_1,_^*∗*^*t*_2_^*∗*^ … *t*_*k*_^*∗*^ is the best solution obtained.

**Figure 3:**
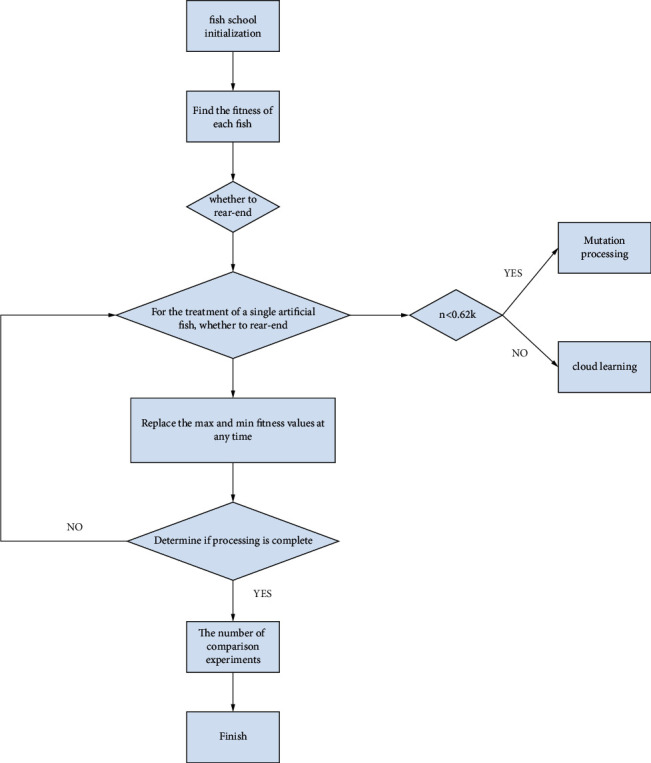
Flowchart of threshold map optimization for artificial fish swarms.

The threshold image segmentation in the artificial fish school is calculated by the maximum value of the entropy value function of the artificial fish school in the original gray image. The threshold value map optimization process of the artificial fish school is shown in Figure 3.

1. Fish swarm initialization. Suppose that the number of fish schools is *S*, the number of experiments is *C*, the fish school detection distance is *D*, the step size is *L*, and *θ* is the proportion of fish schools in the artificial pool.
2. Calculate the fitness of each fish in the fish group by calculating the entropy function, and arrange the fitness values in order, from small to large; and change the information value at any time, and keep abreast of the dynamic maximum *F*_max_ and minimum *F*_min_ adaptive value.
3. Randomly perceive the surrounding of the *n*th artificial fish, find the optimal artificial fish, and judge whether there is a rear-end relationship between the two. If not, go directly to the next step. If there is, follow the rear-end execution.
4. Perform processing *W*_*n*_ on a single artificial fish; if the *n* value is lower than 0.62 k, perform cloud mutation processing on the fish, and if it is greater than 0.62 k, perform a cloud learning phase.
5. Replace the maximum and minimum fitness values and update them on the dynamic board at any time.
6. After processing all the individual fish, go to the next step. If not, continue to repeat the previous steps.
7. Compare the number of updates with the set number of experiments. If the number of updates is greater than the set number, the algorithm terminates; otherwise, the second step is performed. Continue to find the fitness of the fish.

## 4. Simulation Experiments

In order to verify the effectiveness of the method, the paper uses different emphases as the experimental objects and compares the artificial fish swarm algorithm with particle swarm and genetic algorithm. The traditional motion simulation uses the particle swarm computing method. The experimental equipment and materials were the following: computer with Windows 10, Intel 10, Core(TM) i8-3632QM CPU, and 8G running memory, and the translation tool was Matlab (R2017b). Through many experiments, the effectiveness of the image entity simulation image under human motion, the segmentation of the image processing, the number of core points, and the noise reduction effect are compared. The experimental data are as follows.

The two algorithms to be compared need to be compared through a threshold before calculation. In order to ensure consistency, the threshold is uniformly set to 3 in this paper. The reference data obtained in the literature are obtained after experiments and have practical significance. The data sheet is shown in Table 1.

### 4.1. Captured Moving Images

**Figure 4:**
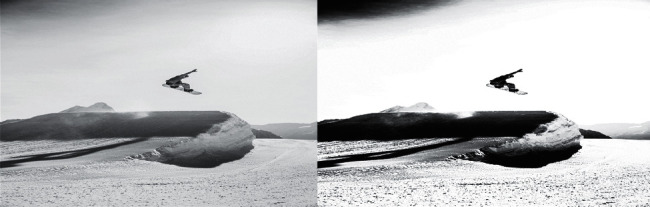
Captured image comparison.

In the first step, an image of human motion needs to be taken, because the original color hierarchy of the background image and the foreground image is easier to distinguish, so the image is used as a contrast image. The comparison of the captured images is shown in Figure 4.

The comparison in Figure 4 shows that the particle swarm algorithm has no way to clear the background and target of the shooting, and there is chromatic aberration, and the background processing is simple. The fish swarm algorithm can see obvious foreground, background, and color, and, in Figure 4, the distinction of brightness is also more obvious.

### 4.2. Monotone Background Image

**Figure 5:**
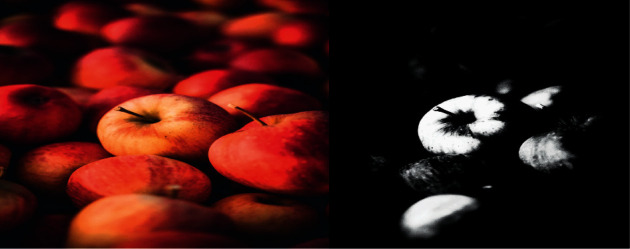
Apple image segmentation comparison.

As shown in Figure 5, the apple image of 512 × 384 is selected because the background is relatively monotonous, and the foreground and background are easier to separate when pixels are separated. From the comparison of the figures, it can be seen that the particle swarm algorithm does not distinguish the background and the foreground clearly, and it is basically impossible to see it. However, the image after the fish swarm algorithm can save the original image information, and, for the information expression of the foreground edge, the particle swarm algorithm is not easy to distinguish between the foreground and the background, which is not conducive to the expression of the internal and edge information of the image.

**Figure 6:**
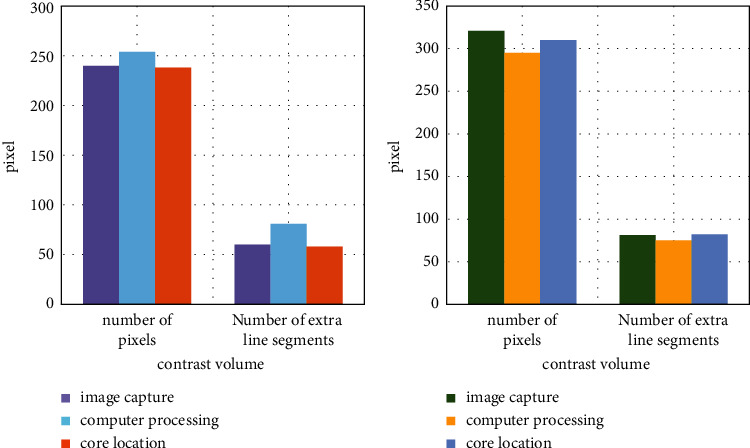
Core point comparison of pixel points.

For the physical simulation of human motion, the arrangement of the pixel points is arranged according to certain rules. However, in the physical simulation of traditional sports, there will be redundant line segments, the core points of the pixel points are judged by the number of line segment connections, and the number of core points is judged by the data of the two physical simulations, as shown in Figure 6.

Through comparison, it is found that the pixel core point of the traditional entity simulation is lower than that based on the fish swarm algorithm, and it is increased by about 35% on the traditional basis. The image obtained in this way is also more vivid, and there is no rough room. The core point reflects the key position of sports. The more core points, the more accurate the capture of sports.

**Figure 7:**
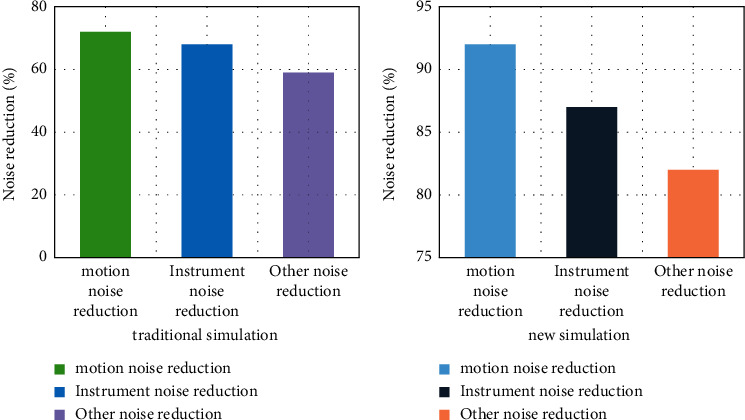
Comparison of traditional simulation and new noise reduction.

When capturing moving images, it is necessary to remove the external sound and then better input into the system for information processing. After processing by the computer, ensure the stability of the computer, because the information with sound is entered into the system will have an impact on the simulation model. The noise reduction comparison between the two is shown in Figure 6. The simulation paper based on the fish swarm algorithm becomes a new type of simulation, as shown in Figure 7.

By comparing the noise reduction effect between the two, it is found that the simulation calculation based on the fish swarm algorithm is 20.3% higher compared to the traditional simulation. At the same time of simulation, it also restores the real motion, well preserves the integrity of the information input to the computer, and also has a good effect on noise reduction of other effects and has obvious optimization in traditional simulation technology.

## 5. Conclusions

In this paper, the physical simulation of traditional sports is compared through sports based on fish swarm algorithm. Through the shortcomings of traditional simulation, the moving entity simulation technology of fish swarm algorithm is introduced. By comparing the effectiveness of the simulated image under human motion, the segmentation of the image processing, the number of core points for the 3D model, and the noise reduction of the two in computer processing, the results found that the physical simulation based on the fish swarm algorithm is more restored on the image, and the captured figures are more segmentable, and the more core points, the more accurate the 3D model, and the noise reduction effect is also improved by 20.3%. The inadequacy of this paper is that the physical simulation is not verified by other algorithms, and only the fish swarm algorithm is verified in the physical simulation. The data referenced in this article also has a certain year, and there may be some errors in the experimental data. With the gradual development of current science and technology, the simulation technology for moving entities will become more mature, and more and more suitable algorithms will be used in entity simulation. The obtained data will also be more accurate, and the influence of external factors on the simulation entity can be excluded. This paper studies the physical simulation of sports, which has certain reference significance for the physical simulation technology.

## Figures and Tables

**Table 1:** Initial parameter table.

Evolutionary algorithms	Parameter settings				
CT-AFSA	*S* = 22	*C* = 100	*D* = 12	*L* = 10	*θ* = 0.75
Related data 1	*S* = 22	*C* = 100	*W* (min) = 0.38	*W* (max) = 0.92	Expansion factor
Related data 2	*S* = 22	*C* = 100	Code length = 10	Crossover probability = 0.65	Mutation probability = 0.1

## Data Availability

The data used to support the findings of this study are available from the author upon request.
